# Greater haemodialysis exposure (‘quotidian haemodialysis’) has different mortality associations by patient age group

**DOI:** 10.1093/ckj/sfae103

**Published:** 2024-04-09

**Authors:** Matthew A Roberts, Christopher E Davies, Leanne Brown, Su Jen Chua, Georgina Irish, Lukas Kairaitis, Rathika Krishnasamy, Emily See, David Semple, Nigel D Toussaint, Andrea K Viecelli, Kevan R Polkinghorne

**Affiliations:** Eastern Health Clinical School, Monash University, Box Hill, Victoria, Australia; Faculty of Health and Medical Science, Adelaide Medical School, Adelaide, South Australia, Australia; Australia and New Zealand Dialysis and Transplant Registry, South Australian Health and Medical Research Institute, Adelaide, South Australia, Australia; Murtupuni Centre for Rural and Remote Health & Australian Institute of Tropical Health and Medicine, James Cook University, Cairns, Queensland, Australia; School of Nursing and Midwifery, Griffith University Brisbane, South Bank, Queensland, Australia; Department of Nephrology, Alfred Health, Prahran, Victoria, Australia; Australia and New Zealand Dialysis and Transplant Registry, South Australian Health and Medical Research Institute, Adelaide, South Australia, Australia; Central and North Adelaide Renal and Transplant Service, Royal Adelaide Hospital, Adelaide, South Australia, Australia; Department of Renal Medicine, Blacktown Hospital, Blacktown, New South Wales, Australia; School of Medicine, Western Sydney University, Sydney, New South Wales, Australia; Department of Nephrology, Sunshine Coast University Hospital, Birtinya, Queensland, Australia; Faculty of Medicine, University of Queensland, Brisbane, Queensland, Australia; Department of Nephrology, Royal Melbourne Hospital, Parkville, Victoria, Australia; Department of Critical Care, University of Melbourne, Melbourne, Victoria, Australia; Department of Intensive Care, Royal Melbourne Hospital, Parkville, Victoria, Australia; Department of Nephrology, Royal Children's Hospital, Parkville, Victoria, Australia; Department of Renal Medicine, Auckland District Health Board, Auckland, New Zealand; Faculty of Medical and Health Sciences, University of Auckland, Auckland, New Zealand; Department of Nephrology, Royal Melbourne Hospital, Parkville, Victoria, Australia; Department of Medicine, University of Melbourne, Parkville, Victoria, Australia; Department of Kidney and Transplant Services, Princess Alexandra Hospital, Brisbane, Queensland, Australia; Australasian Kidney Trials Network, University of Queensland, Brisbane, Queensland, Australia; Department of Medicine, Southern Clinical School, Monash University, Melbourne, Victoria, Australia; School of Public Health and Preventive Medicine, Monash University, Melbourne, Victoria, Australia; Department of Nephrology, Monash Health, Clayton, Victoria, Australia

**Keywords:** haemodialysis, mortality, quotidian dialysis, session frequency, treatment time

## Abstract

**Background:**

Worldwide, most people requiring kidney replacement therapy receive haemodialysis (HD) three times per week. Greater HD time and/or frequency may improve survival, but implementation requires understanding potential benefits across the range of patients.

**Methods:**

Using data from the Australia and New Zealand Dialysis and Transplant Registry, we assessed whether quotidian HD (defined as >3 sessions/week and/or >5 h/session) was associated with reduced mortality in adult patients. The primary outcome of all-cause mortality was analysed by a time-varying Cox proportional hazards model with quotidian HD as the exposure of interest.

**Results:**

Of 24 138 people who received HD between 2011 and 2019, 2632 (10.9%) received quotidian HD at some stage. These patients were younger, more likely male and more likely to receive HD at home. Overall, quotidian versus standard HD was associated with a decreased risk for all-cause mortality {crude hazard ratio [HR] 0.50 [95% confidence interval (CI) 0.45–0.56]}, but an interaction between quotidian HD and age was identified (*P* = .005). Stratified by age groups and splitting follow-up time where proportional hazards were violated, the corresponding HR compared with standard HD was 2.43 (95% CI 1.56–3.79) for people >75 years of age in the first year of quotidian HD, 1.52 (95% CI 0.89–2.58) for 1–3 years and 0.95 (95% CI 0.51–1.78) for ≥3 years. There was no significant survival advantage in younger people.

**Conclusions:**

Although quotidian HD conferred survival benefit in crude analyses, people ≥75 years of age had greater mortality with quotidian HD than standard HD. The mortality benefit in younger people was attenuated when adjusted for known confounders.

## Graphical Abstract

**Graphical Abstract:**
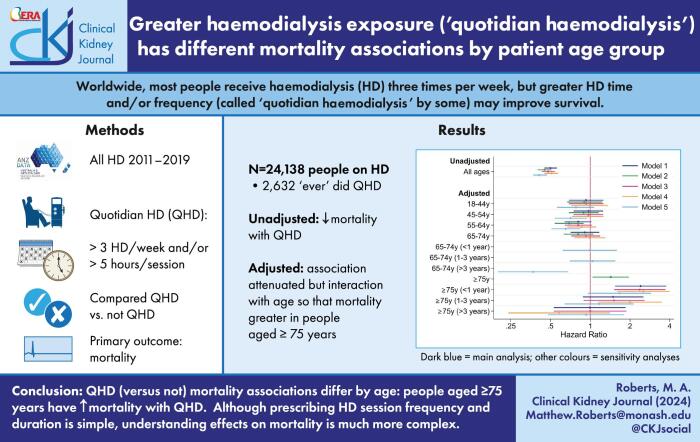

KEY LEARNING POINTS **What was known:**

1. The term quotidian haemodialysis (HD) was introduced in some registries to classify prescription of HD sessions and hours in order to study mortality and other associations.
2. Studies of this association have been inconclusive to date.

**This study adds:**

1. This study shows that the reporting of associations of HD sessions and hours with mortality is confounded by measured and (likely) unmeasured factors and by effect modification by age.
2. This study describes some but not all of these factors.

**Potential impact:**

1. While quotidian HD appears to be associated with decreased mortality overall, many factors need to be considered in individual patients to decide who will benefit and who will not.
2. Collecting data in registries to study HD hours and sessions requires clear and implementable definitions and terminology for dialysis prescriptions and data that capture patient and health system factors that determine this prescription.

## INTRODUCTION

Haemodialysis (HD) is a treatment for people with kidney failure intended to maintain life, reduce symptoms and prevent complications. Session frequency and treatment time are critical components of the HD prescription. Most patients undergoing HD worldwide receive 3–4 h of HD three times per week [1], with great variability. In the USA, 60% of patients on HD receive <225 min/session, whereas 60% of Australian and New Zealand patients receive >250 min/session [2]. Observational studies suggest that longer treatment time is associated with improvement in nutrition, left ventricular hypertrophy, hypertension, anaemia and calcium–phosphate balance [3, 4] and better survival [5, 6], observations reported very early in the history of dialysis [7].

Although the dictionary definition of quotidian is ‘daily’, the term quotidian dialysis, first used in 2004 [8], describes HD more frequent and/or longer in duration than conventional thrice-weekly HD. The International Quotidian Dialysis Registry performed two analyses comparing registry participants receiving quotidian HD with Dialysis Outcomes Practice Patterns Study (DOPPS) participants receiving conventional HD [9, 10]. Using propensity score matching to compare Frequent Hemodialysis Network (FHN) trial participants with DOPPS participants on standard HD, the FHN participants receiving short (predominantly <3 h/session) daily (usually 5–6 sessions/week) HD experienced higher mortality [10], whereas FHN participants receiving long (mean 7.4 h/session) daily (mean 4.8 sessions/week) HD experienced reduced mortality [9]. The centre-based short daily FHN trial demonstrated a favourable outcome for frequent HD for the two co-primary composite outcomes of death plus left ventricular mass or health-related quality of life, but there were too few deaths to assess mortality [11]. Therefore, the impact of more frequent HD on patient survival is uncertain.

We tested the hypothesis that patients who received quotidian HD in the Australia and New Zealand Dialysis and Transplant (ANZDATA) Registry had greater survival than those who received non-quotidian HD. We also assessed whether there was any association between quotidian HD and mortality by age, patient size and performing HD at home.

## MATERIALS AND METHODS

### Study design and population

This observational cohort study included all adult patients (≥18 years of age) who commenced kidney replacement therapy (KRT) in Australia and New Zealand from 1 January 2011 through 31 December 2019 and received HD for at least 1 day. We excluded patients who received KRT outside of Australia or New Zealand during the study period. The Eastern Health Human Research Ethics Committee approved the study (LR22-074-92329).

### Exposure

The primary exposure of interest was quotidian HD. The ANZDATA Registry introduced a treatment code for quotidian HD in 2011 that defined quotidian HD as >3 sessions/week, >5 h/session or both. A treatment code (Supplemental Table S1) recorded the date of starting and ending quotidian HD, enabling exposure time to be measured. This relied on centres recognizing and reporting when a patient met the above criteria. The ANZDATA Registry only collected HD sessions/week and treatment time in the annual survey. The comparator of non-quotidian HD will be referred to as standard HD.

### Outcomes

The primary outcome was all-cause mortality. A secondary outcome was the time patients received quotidian HD, defined as the time from starting quotidian HD to transfer from quotidian to standard HD or another treatment code. The cohort used for the outcome of time on quotidian HD was the ‘ever quotidian’ group from the comparison of baseline characteristics.

### Study variables

Study variables collected by the registry were included in multivariable analyses if considered potential confounders. These included variables that were associated with receiving quotidian HD or not and associated with mortality: age at KRT start, body mass index (BMI) at KRT start, location of HD (home versus facility at first HD or at first quotidian HD if ever had quotidian HD), vascular access at first HD (arteriovenous fistula or graft versus catheter), primary cause of kidney disease, late referral (initial review by nephrologist <3 months before the start of KRT), smoking status (current, former or never), Australian jurisdiction or New Zealand, ethnicity (Caucasian, Aboriginal and Torres Strait Islander, Asian, Māori, Pacific Islander or other/not reported), diabetes status (including insulin-dependent and non-insulin-dependent diabetes), cerebrovascular disease, peripheral vascular disease, coronary artery disease and chronic lung disease at KRT start. These last four were considered present if reported as ‘yes’ or ‘suspected’. None of these variables were considered in the causal pathway between the type of HD and survival.

### Reliability of reporting of quotidian HD treatment code variable

We assessed the reliability of the quotidian HD treatment code variable by examining the number of switches from or to quotidian HD over the calendar year to identify potential ‘dummy dates’ assigned when the true date was uncertain. We verified whether people performing quotidian HD were performing hours and sessions consistent with the annual survey (current ANZDATA survey form: https://www.anzdata.org.au/wp-content/uploads/2023/04/ANZDATADialysisAndTransplantSurvey.pdf).

### Statistical analysis

Baseline characteristics were compared between patients who ever had quotidian dialysis for at least 1 day by the treatment code and those who never received quotidian dialysis (standard HD). Parametric and non-parametric tests were used to compare baseline characteristics between quotidian and standard HD.

Survival analysis was performed with exposure to quotidian or standard HD potentially varying over time, determined using the treatment code variable. People exposed to quotidian or standard HD were censored at transfer to peritoneal dialysis (PD) for ≥30 days, kidney transplantation, recovery of kidney function, loss to follow-up or on 31 December 2019. Deaths within 90 days of transfer to standard HD were included as quotidian HD deaths to account for potentially informative transfers from quotidian to standard HD.

Cox proportional hazards regression models were used with quotidian or standard HD as a time-varying covariate. Crude and adjusted analyses are reported with adjustment for all study variables defined above. Age and BMI were categorized as 18–44, 45–54, 55–64, 65–74 and ≥75 years and <18.5, 18.5–<25, 25–<30 and ≥30 kg/m^2^, respectively. Interactions of quotidian dialysis with age, BMI and location of dialysis were evaluated because they may influence the relationship between dialysis prescription and survival. Diabetes status was included in the adjusted models as a comorbidity (not primary kidney disease). Patients with missing values on any covariates were excluded from adjusted analyses. Testing for proportional hazards was done using Schoenfeld residuals on the full adjusted model.

Sensitivity analyses were performed to test the robustness and consistency of our findings with changing assumptions. In model 2, death was attributed to modality at the time of death and not within 90 days of transfer. In model 3, a Cox proportional hazard model with shared frailty using a random effects term for centre was performed to assess whether adjusting for centre-level variation changed results. Two further models assessed the effect of exclusion of patients with possibly incorrect dates of starting or stopping quotidian HD (model 4) and the results with classification of quotidian or standard HD relying on the annual survey and not the ‘real-time’ treatment code variable (model 5).

Analyses were performed using Stata version 16.1 (StataCorp, College Station, TX, USA).

## RESULTS

### Baseline characteristics

**Figure 1:**
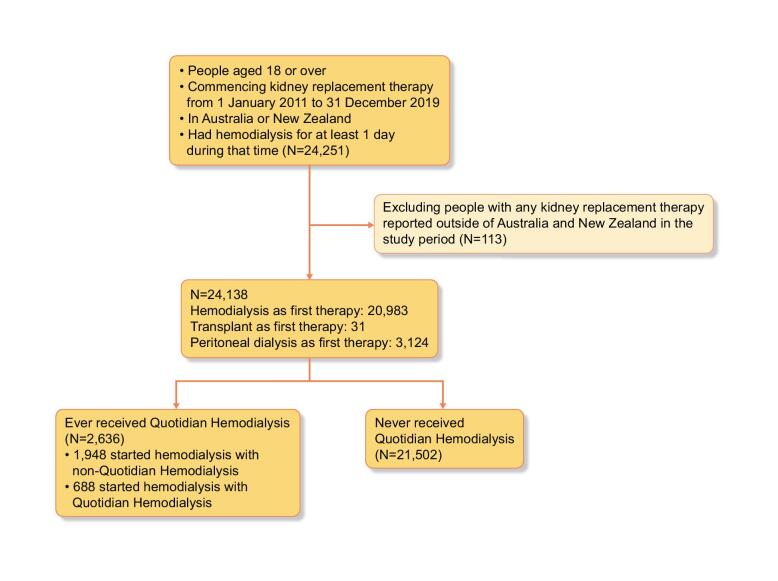
Study flow diagram.

There were 24 251 patients ≥18 years of age reported to the ANZDATA Registry who commenced KRT between 1 January 2011 and 31 December 2019 and received HD for at least 1 day. After exclusions, 2632 (10.9%) of the 24 138 remaining patients received quotidian HD at any stage, the majority having standard HD first (Fig. 1).

Patients who received quotidian HD were younger, more often male, more likely to have dialysis at home and had a higher BMI compared with patients who never received quotidian HD (Table 1). At the first annual survey after commencing quotidian HD, 1123 (43%) received >3 sessions/week, 832 (32%) >5 h/session, 396 (15%) met both criteria and 285 (11%) did not meet the quotidian HD definition (see the Reliability section). Patients having quotidian HD at home were more likely to receive increased sessions/week than those having quotidian HD at a facility (Supplementary Table S2).

**Table 1:** Baseline characteristics of patients by quotidian dialysis status. For the ever quotidian group, baseline is the start of quotidian dialysis, whereas for the never quotidian group baseline is the start of HD.

Characteristic	Ever quotidian HD (*n* = 2636)	Never quotidian HD (*n* = 21 502)
Demographics		
Age (years), mean ± SD	54.3 ± 13.8	61.8 ± 14.7
Age category (years)		
18–44	614 (23)	2801 (13)
45–54	663 (25)	3385 (16)
55–64	731 (28)	4938 (23)
65–74	454 (17)	5800 (27)
≥75	174 (7)	4578 (21)
Male, *n* (%)	1959 (74)	13 100 (61)
BMI category, *n* (%)		
Underweight	17 (1)	518 (3)
Normal	475 (18)	5759 (28)
Overweight	612 (24)	6384 (31)
Obese	1475 (57)	8066 (39)
Country, *n* (%)		
Australia	2013 (76)	18 426 (86)
New Zealand	623 (24)	3076 (14)
Ethnicity, *n* (%)		
Caucasian	1540 (58)	13 312 (62)
Aboriginal and Torres Strait Islander	161 (6)	2403 (11)
Asian	214 (8)	1907 (9)
Māori	291 (11)	1168 (5)
Pacific Islanders	255 (10)	1296 (6)
Other/not reported	175 (7)	1416 (7)
HD location: home at first HD or first quotidian HD if quotidian (versus facility)	1163 (44)	236 (1)
Vascular access at first HD, *n* (%)		
Arteriovenous fistula or graft	1443 (56)	7987 (38)
Central catheter	1119 (44)	12 775 (62)
Late referral, *n* (%)	424 (16)	4420 (21)
Baseline HD after first transplant, *n* (%)	34 (1.3)	59 (0.3)
Baseline HD after first PD, *n* (%)	439 (17)	2800 (13)
Primary kidney disease, *n* (%)		
Diabetes	1000 (38)	9150 (43)
Glomerular disease	613 (23)	3597 (17)
Hypertension	269 (10)	2833 (13)
Polycystic kidney disease	238 (9)	1006 (5)
Reflux nephropathy	67 (3)	309 (1)
Other/uncertain/not reported	449 (17)	4607 (21)
Smoking status, *n* (%)		
Current	335 (13)	2778 (13)
Former	1128 (43)	8436 (40)
Never	1133 (44)	9761 (47)
Comorbidities, *n* (%)		
Diabetes	1312 (50)	11 875 (56)
Coronary artery disease	817 (31)	8376 (39)
Cerebrovascular disease	252 (10)	2867 (13)
Peripheral vascular disease	528 (20)	4936 (23)
Chronic lung disease	420 (16)	3738 (18)

Of the quotidian HD group, 174 (7%) were ≥75 years of age. In this group, quotidian HD criteria were met by the sessions/week criterion (47%), hours/session criterion (17%) or both (4%). In 32%, quotidian HD criteria were not met or not recorded (see the Reliability section). Of persons ≥75 years of age doing quotidian HD, 137 (79%) did this at a facility rather than at home (Supplementary Table S2, percentages not shown across rows).

### Primary outcome: survival

**Figure 2:**
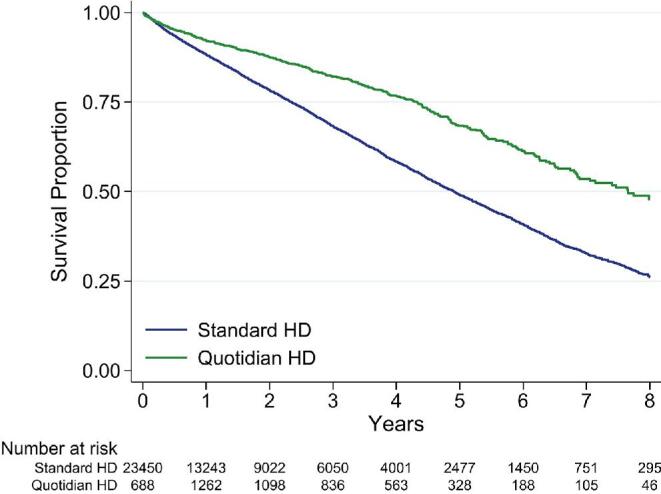
Unadjusted patient survival by time-varying exposure to quotidian HD versus standard HD in the study cohort. The numbers at risk below the graph are the number doing quotidian and standard HD at that time point. Because it is a time-varying analysis, the Quotidian HD group increases between time 0 and 1 year.

**Figure 3:**
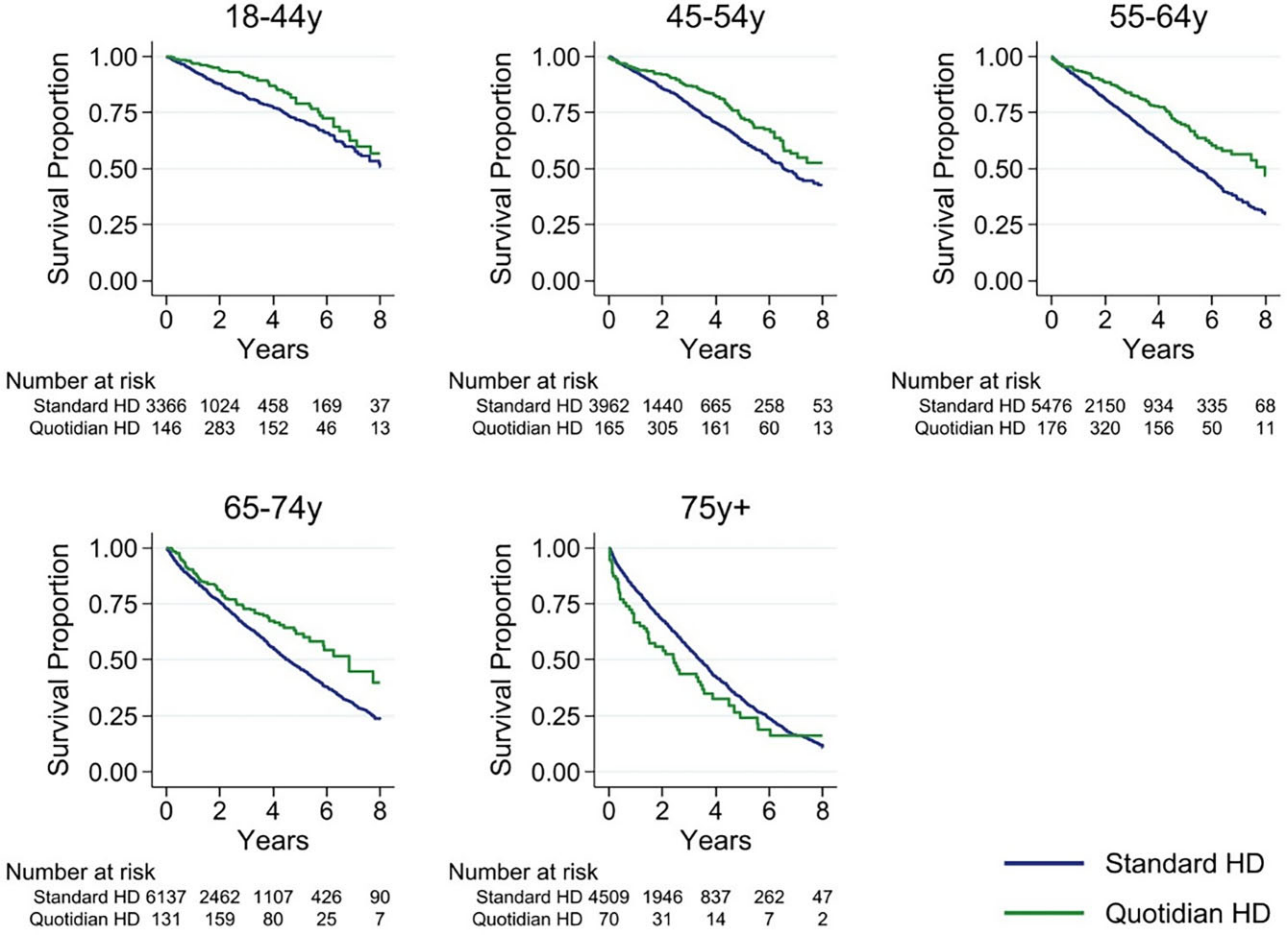
Unadjusted patient survival by age group and time-varying exposure to quotidian HD in the study cohort versus standard HD.

Over a median follow-up of 1.5 years/patient [interquartile range (IQR) 0.44–3.33) and a total of 52 227 person-years, 6905 deaths occurred. In unadjusted time-varying analysis, people treated with quotidian HD had superior survival compared with people not exposed to quotidian HD (Fig. 2). Overall, 1-year unadjusted survival was 92.2% [95% confidence interval (CI) 90.4–93.6] for people who received quotidian HD and 88.3% (95% CI 87.8–88.7) with standard HD; 5-year survival was 68.4% (95% CI 65.0–71.4) and 49.0% (95% CI 48.0–50.1), respectively. The crude mortality hazard ratio (HR) was 0.50 (95% CI 0.45–0.56). In stratified analyses, survival was superior for quotidian HD for every age group except people ≥75 years of age (Table 2, Fig. 3).

**Table 2:** Crude 1-year and 5-year survival by age subgroups comparing time-varying exposure to quotidian HD versus standard HD.

	1-year survival, % (95% CI)		5-year survival, % (95% CI)	
Age subgroup (years)	Quotidian HD	Standard HD	Quotidian HD	Standard HD
18–44	96.8 (93.9–98.3)	93.8 (92.7–94.7)	79.0 (72.3–84.3)	71.6 (68.4–74.6)
45–54	94.7 (91.2–96.8)	93.2 (92.2–94.0)	72.5 (65.9–78.1)	62.3 (59.5–65.1)
55–64	93.5 (89.9–95.9)	90.4 (89.4–91.2)	69.1 (62.5–74.8)	53.4 (51.1–55.7)
65–74	89.8 (84.7–93.3)	86.2 (85.2–87.2)	61.5 (53.0–68.9)	46.2 (44.2–48.1)
≥75	66.6 (54.1–76.4)	81.3 (80.1–82.5)	24.2 (13.3–36.9)	32.4 (30.4–34.3)

**Figure 4:**
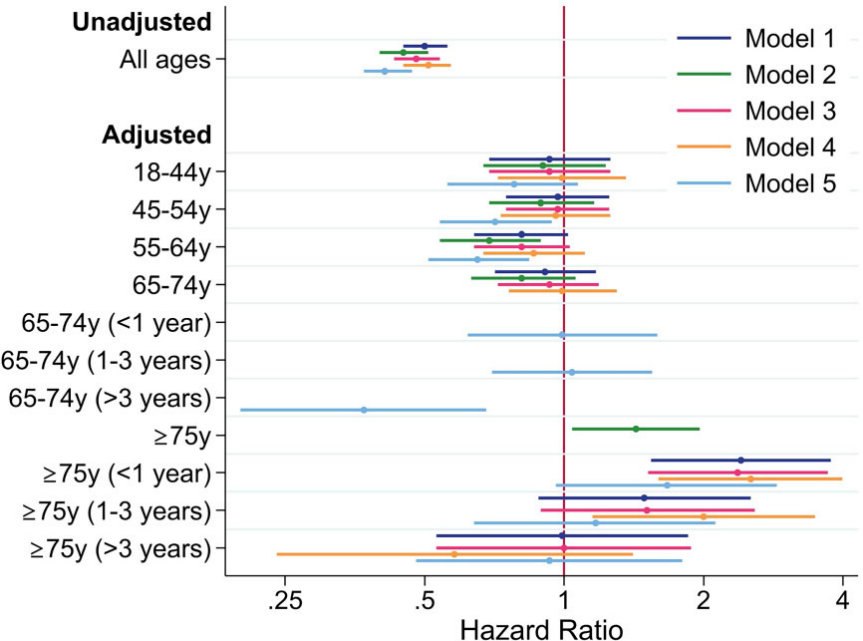
HR of death for quotidian versus standard HD stratified by age and adjusting for variables outlined in the Methods. Main model (model 1): analysis using time-varying exposure to HD type, assigning exposure to dialysis type up to 90 days before death; model 2: analysis using time-varying exposure to HD type, but assigning exposure to dialysis type at time of death; model 3: model 1 with treatment centre as a random effect using a shared frailty model; model 4: model 1 with exclusion of patients with potentially incorrect dates of starting or stopping quotidian HD; model 5: model 1, but with the classification of quotidian or standard HD relying on the annual survey and not the real-time treatment code variable. Where hazards were not proportional, the group was divided into <1 year, 1–3 years and >3 years from dialysis start. See Supplementary Table S3 for actual HRs.

In a multivariable model adjusting for covariates listed in the Methods section, 2858 (11.8%) patients were excluded due to missing variables. The interaction term for quotidian HD and age was significant (*P* = .002) but interactions with BMI category or HD location were not (*P* > .05). Due to non-proportional hazards, we separated follow-up time to analyse the effect of variables separately at <1 year, 1–<3 years and ≥3 years. In persons ≥75 years of age there was a 2.4-fold increased hazard of death for quotidian HD versus standard HD in the first year of HD that was lower at 1–3 years and absent after 3 years (Fig. 4 model 1, Supplementary Table S3). The risk of death with quotidian versus standard HD was not significantly different in younger patients.

Collapsing the ≥75-year-old patients into age ≥65 years attenuated the HR for death in the first year of dialysis [HR 1.37 (95% CI 1.06–1.76)], which was not stratified by age group because the age–quotidian HD interaction was not significant (*P* = .17). In separate analyses of patients by cardiovascular comorbidity status, people ≥75 years of age without cardiovascular comorbidity had a greater hazard of death that did not vary over time [HR 2.21 (95% CI 1.30–3.77)], whereas the age–quotidian HD interaction was not significant for those with cardiovascular comorbidity (*P* = .13), and the non-age-stratified HR was 1.45 (95% CI 1.09–1.94) in the first year.

### Sensitivity analyses using alternate models

Ignoring informative censoring demonstrated increased mortality with quotidian HD in people ≥75 years of age and decreased mortality in people 55–64 years of age (Fig. 4 model 2, Supplementary Table C3). Excluding treatment centres with <50 patients receiving HD, the proportion of people receiving HD at each centre who ever received quotidian HD ranged from 0 to 40% (Supplementary Figure S1). Including treatment centre as a random effect in a shared frailty model gave results similar to model 1 (Fig. 4 model 3, Supplementary Table S3). Excluding 301 patients with exposure based on potentially unreliable dates (see below), quotidian HD was associated with increased mortality in people ≥75 years of age over the first 3 years of HD (Fig. 4 model 4, Supplementary Table S3). Classifying HD status at the annual survey instead of by treatment codes demonstrated decreased mortality with quotidian HD in people 45–64 years of age but no increased mortality in people ≥ 75 years of age (Fig. 4 model 5, Supplementary Table S3). The interaction term for age and quotidian HD was significant for all models (model 2: *P* = .01; model 3: *P* = .003; model 4: *P* < .001; model 5: *P* = .009).

### Secondary outcome: time on quotidian HD

The median time to commencing quotidian HD after the first HD was 3.9 months (IQR 0.0–13.2); the longest time was 7.9 years. Overall, people underwent quotidian HD for a median of 3.0 years (IQR 1.0–6.0). This was 4.3 years (IQR 1.5–6.9) in people 18–44 years of age and 0.8 years (IQR 0.2–2.0) in people ≥75 years of age. The duration of quotidian HD was longer if quotidian HD was first performed at home [median 4.1 years (IQR 1.8–7.0)] compared with in a facility [2.1 years (IQR 0.6–5.3)].

Reasons for discontinuation of quotidian dialysis were return to standard HD or PD for ≥30 days (33%), kidney transplantation (22%), death (12%) and return of native kidney function (1%). At the end of the study, 32% were still receiving quotidian HD.

### Reliability of the quotidian treatment code variable

There were 2975 changes of prescription to or from quotidian HD identified by the treatment code. The median number of switches to or from quotidian HD per calendar date was 7, but three dates substantially exceeded this number: 1 January (*n* = 32), 15 June (*n* = 309) and 31 December (*n* = 35). These were likely ‘dummy dates’ entered when the exact date was unknown and thus 12% of switches may have incorrect dates.

In 285 (11%) patients classified as quotidian HD by the treatment code variable, hours and sessions at the subsequent annual survey were inconsistent with quotidian HD (*n* = 241) or were missing (*n* = 44). Among people ≥75 years of age who received quotidian HD, 55 (32%) of 174 recorded hours inconsistent with quotidian HD (*n* = 49) or had missing hours (*n* = 6). Of the 241 people in whom hours and sessions at the annual survey were inconsistent with quotidian HD, 176 (73%) commenced quotidian HD but returned to standard HD before the annual survey. Among the 2636 people who received quotidian HD, 21% (36/174) ≥75 years of age received quotidian HD between the annual surveys only, compared with 5.7% (140/2462) in people <75 years of age.

## DISCUSSION

Over a 9-year period, 2636 (10.9% of all HD) people received quotidian HD with the date of commencing and ending this therapy recorded in the ANZDATA Registry. Receiving quotidian HD was associated with better survival in the crude analysis but poorer survival for people ≥75 years of age when stratified by age. A time-varying multivariable analysis demonstrated this interaction between quotidian HD and age was significant. Multivariable adjustment attenuated the association of quotidian HD with better survival in younger patients. Sensitivity analyses using models based on four assumptions did not substantially alter the findings.

This analysis has three implications: receiving quotidian HD was associated with poorer outcomes in people ≥75 years of age; the association of quotidian HD with better survival is confounded by demographics, dialysis-related factors, comorbidities and reason for prescribing quotidian HD; and studies of HD session frequency and duration require careful attention to definitions and terminology to avoid missing data, misclassification and inaccurate exposure times.

The association of quotidian HD with higher mortality in people ≥75 years of age is plausible but likely confounded. One indication for increasing session frequency or duration is to control volume overload in patients with heart failure, a variable not collected by the ANZDATA Registry. The cohort ≥75 years of age was small (7% of those doing quotidian HD), limiting granular subgroup analysis. However, 79% of this cohort received quotidian HD in a facility as opposed to at home, and 21% received quotidian HD for a short period between annual surveys. Thus some people ≥75 years of age could have received quotidian HD during periods of intercurrent illness, contributing to their mortality risk. Ultimately we cannot determine from our data whether the mortality association in this group is a result of the indication for quotidian HD or the treatment itself.

In contrast to people ≥75 years of age, younger people had better survival with quotidian HD, but this was attenuated by multivariable adjustment. Age, BMI [12] and location of HD could all influence survival. The proportion of people doing HD at home was much greater with quotidian versus standard HD, but our analysis could not eliminate the selection bias and residual confounding that contribute to better survival in people having HD at home [13]. Further, it was not possible to do competing risks analysis accounting for being censored before a mortality event (i.e. transplant), as the exposure was time-varying in our analysis.

When the ANZDATA Registry introduced a treatment code variable to measure exposure to above-standard dialysis, the term ‘quotidian dialysis’ was used, consistent in name with the International Quotidian Dialysis Registry [14]. Literal interpretation of the term ‘quotidian dialysis’ may have caused underreporting of dates of change, leading to 12% being ‘dummy dates’. Given the competing priorities of HD unit staff and the complexity of identifying this change, more apt descriptive terminology may have helped. Finding appropriate terminology for above-standard HD is not straightforward. Some investigators have used the term ‘extended HD’ for HD >15 h/week [15], whereas the term ‘expanded HD’ has been used to describe a different aspect of dialysis prescription, i.e. middle cut-off dialysis membranes [16]. Clear and broadly accepted terminology is required in this area.

Although this registry analysis cannot provide definitive recommendations regarding quotidian HD, well-designed prospective randomized controlled trials are unlikely to be successfully performed. Trials to date have suffered difficulties in recruitment, generalizability, adherence to the regimen (particularly the greater number of sessions or hours) and separation of treatment arms. In the FHN Daily Trial, one-third of patients who consented could not be randomized due to personal schedules, travel burden, adherence and clinic capacity [17], and only 8% of eligible patients approached were randomized [11]. The FHN Nocturnal Dialysis Trial recruited 87 of the planned 250 participants [18], with common barriers to home HD impacting recruitment [19]. The Clinical Trial of IntensIVe (ACTIVE) Dialysis Study randomized 200 of 659 eligible patients to >24 h/week HD versus <18 h/week in countries with a greater prevalence of home HD than in North America [20]. Studies of HD above the standard frequency and duration are strongly impacted by doctor, system and patient factors that influence provision of above-standard HD. People recruited to studies differ from most people receiving HD, such as participants in the FHN trials who were 10 years younger than US Renal Data System patients [21]. Adherence of patients who consented to being randomized was lower at 12 months in above-standard HD in the above trials (ranging from 72.7 to 77.7%) than for participants randomized to standard HD (ranging from 94.5 to 97.6%) [11, 18, 20].

In conclusion, quotidian HD according to the ANZDATA definition demonstrated differing mortality associations by age that were susceptible to confounding. Although the number and duration of HD sessions should be a simple part of HD prescription, future studies to determine the sessions and hours to prescribe for best patient outcomes require a deeper understanding of the reasons above-standard HD is prescribed, and easily understood definitions of HD regimens, and must overcome the complex interplay of patient, doctor and health system factors that drive or limit the actual sessions and hours of HD patients.

## Supplementary Material

sfae103_Supplemental_File

## Data Availability

The data underlying this article will be shared upon reasonable request to the corresponding author.
