# Supplementary material for: Greater haemodialysis exposure (‘quotidian haemodialysis’) has different mortality associations by patient age group
Source: Clin Kidney J. 2024 Apr 9;17(5):sfae103. doi: 10.1093/ckj/sfae103 (PMC11210063; doi:10.1093/ckj/sfae103)

Greater hemodialysis exposure ("quotidian hemodialysis") has different mortality associations by patient age group: online data supplement

Contents

Supplementary Table 1. 2

Major treatment codes used in the ANZDATA Registry: these are mutually exclusive and designed to reflect the patient's kidney failure journey. 2

Supplementary Table 2. 3

Comparison of baseline characteristics of home dialysis versus facility dialysis patients at first quotidian dialysis 3

Supplementary Table 3. 6

Hazard Ratio for death for Quotidian versus Standard HD stratified by age and adjusting for variables outlined in the Methods. 6

Supplementary Figure 1. 8

The proportion of HD patients who ever received quotidian dialysis by center for centers with 50 or more patients 8

Supplementary Table 1.

Major treatment codes used in the ANZDATA Registry: these are mutually exclusive and designed to reflect the patient's kidney failure journey.

**ANZDATA Treatment codes**
Standard hemodialysis (also coded as home, hospital or facility)
Quotidian hemodialysis (also coded as home, hospital or facility)
Peritoneal dialysis (also coded as automated or continuous ambulatory)
Transplant
Recovery of kidney function
Loss to follow up
Death

Supplementary Table 2.

Comparison of baseline characteristics of home dialysis versus facility dialysis patients at first quotidian dialysis

**Characteristic**	**Home** n=1163	**Facility** n=1473	**p-value**
Reason meets quotidian definition according to first Annual Survey with quotidian sessions or hours reported			<0.001
>3 sessions/week	662 (57%)	461 (31%)	
>5 hours per session	212 (18%)	620 (42%)	
>3 sessions/week and >5 hours per session	235 (20%)	161 (11%)	
Missing data	54 (5%)	231 (16%)	
Demographics			
Age (mean±SD)	52.8±12.8	55.6±14.5	<0.001
Age Category:			<0.001
18-44 years	289 (25%)	325 (22%)	
45-54 years	329 (28%)	334 (23%)	
55-64 years	337 (29%)	394 (27%)	
65-74 years	171 (15%)	283 (19%)	
≥75 years	37 (3%)	137 (9%)	
Sex (male)	880 (76%)	1079 (73%)	0.16
BMI Category (missing n=56, 2%)			0.065
Underweight	6 (1%)	11 (1%)	
Normal	189 (17%)	286 (20%)	
Overweight	263 (23%)	349 (24%)	

Obese	683 (60%)	793 (55%)	
Country			<0.001
Australia	829 (71%)	1184 (80%)	
New Zealand	334 (29%)	289 (20%)	
Ethnicity			0.19
Caucasian	679 (58%)	861 (58%)	
Aboriginal and Torres Strait Islander	71 (6%)	90 (6%)	
Asian	81 (7%)	133 (9%)	
Maori	145 (12%)	146 (10%)	
Pacific Islanders	109 (9%)	146 (10%)	
Other/Not reported	78 (7%)	97 (7%)	
Dialysis variables			
Vascular access at first HD (missing n=74, 3%)			<0.001
Arteriovenous fistula or graft	737 (65%)	706 (49%)	
Central catheter	397 (35%)	722 (51%)	
Late referral (yes, missing n=37, 1%)	131 (11%)	293 (20%)	<0.001
Baseline HD after first transplant	19 (2%)	15 (1%)	0.16
Baseline HD after first Peritoneal dialysis	198 (17%)	241 (16%)	0.65
Primary kidney disease			<0.001
Diabetes	410 (35%)	590 (40%)	
Glomerular Disease	295 (25%)	318 (22%)	
Hypertension	98 (8%)	171 (12%)	
Polycystic kidney disease	129 (11%)	109 (7%)	

Reflux nephropathy	34 (3%)	33 (2%)	
Other/Uncertain/Not reported	197 (17%)	252 (17%)	
Co-morbidities			
Smoking Status (missing n=40, 2%)			0.16
Current	135 (12%)	200 (14%)	
Former	496 (43%)	632 (44%)	
Never	522 (45%)	611 (42%)	
Diabetes (missing n=11, 0.4%)	541 (47%)	771 (53%)	0.003
Coronary artery disease (missing n=9, 0.3%)	290 (25%)	527 (36%)	<0.001
Cerebrovascular disease (missing n=9, 0.3%)	80 (7%)	172 (12%)	<0.001
Peripheral vascular disease (missing n=10, 0.4%)	167 (14%)	361 (25%)	<0.001
Chronic Lung Disease (missing n=10, 0.4%)	169 (15%)	251 (17%)	0.08

Supplementary Table 3.

Hazard Ratio for death for Quotidian versus Standard HD stratified by age and adjusting for variables outlined in the Methods.

These are the hazard ratios in **Figure 4**. Main Model (Model 1) = analysis using time-varying exposure to HD type, assigning exposure to dialysis type up to 90 days before death; Model 2 = analysis using time-varying exposure to HD type, but assigning exposure to dialysis type at time of death.; Model 3 = Model 1 with treatment center as random effect using a shared frailty model; Model 4 = Model 1 with exclusion of patients with potentially incorrect dates of starting or stopping quotidian HD; Model 5 = Model 1 but with classification of quotidian or standard HD relying on the Annual Survey and not the "real time" treatment code variable. Where hazards were not proportional, the group has been divided into <1 year, 1 to 3 years and >3 years from dialysis start.

Age Group	Quotidian compared with Non-quotidian [HR (95%CI)]				
	Model 1	Model 2	Model 3	Model 4	Model 5
All ages, unadjusted	0.50 (0.45, 0.56)	0.45 (0.40, 0.51)	0.48 (0.43, 0.54)	0.51 (0.45, 0.57)	0.41 (0.37, 0.47)
Adjusted model by age category					
18 to 44 years	0.93 (0.69, 1.26)	0.90 (0.67, 1.23)	0.93 (0.69, 1.26)	0.99 (0.72, 1.36)	0.78 (0.56, 1.07)
45 to 54 years	0.97 (0.75, 1.25)	0.89 (0.69, 1.16)	0.97 (0.75, 1.25)	0.96 (0.73, 1.26)	0.71 (0.54, 0.94)

55 to 64 years	0.81 (0.64, 1.02)	0.69 (0.54, 0.89)	0.81 (0.64, 1.03)	0.86 (0.67, 1.11)	0.65 (0.51, 0.84)
65 to 74 years	0.91 (0.71, 1.17)	0.81 (0.63, 1.06)	0.93 (0.72, 1.19)	0.99 (0.76, 1.30)	
65 to 74 years (<1 year)					0.99 (0.62, 1.59)
65 to 74 years (1 to 3 years)					1.04 (0.70, 1.55)
65 to 74 years (>3 years)					0.37 (0.20, 0.68)
≥75 years		1.43 (1.04, 1.96)			
≥75 years (<1 year)	2.41 (1.54, 3.76)		2.37 (1.52, 3.71)	2.53 (1.60, 3.99)	1.67 (0.96, 2.88)
≥75 years (1 to 3 years)	1.49 (0.88, 2.53)		1.51 (0.89, 2.58)	2.00 (1.15, 3.48)	1.17 (0.64, 2.12)
≥75 years (>3 years)	0.99 (0.53, 1.85)		1.00 (0.53, 1.88)	0.58 (0.24, 1.41)	0.93 (0.48, 1.80)

Supplementary Figure 1.

The proportion of HD patients who ever received quotidian dialysis by center for centers with 50 or more patients

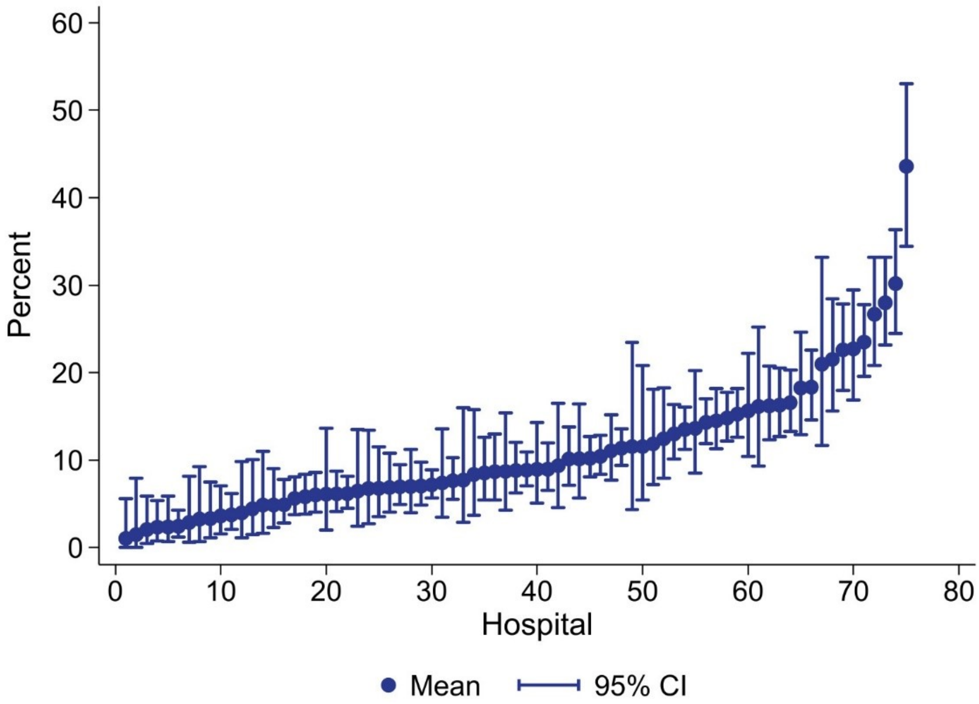

Supplement: sfae103_Supplemental_File [file sfae103_Supplemental_File.pdf]
